# Mercury in Typhoid Fever

**Published:** 1857-03

**Authors:** 


*Mercury in Typhoid Fever.*

The following letter should have received attention earlier, but it had been accidentally overlooked.—ED.

CHARLESTON, ILL. Jan. 12, 1857.

*Editor North-Western Medical and Surgical Journal:—*

The profession have been furnished liberally with one fact connected with the Pathology of Typhoid Fever, which, it occurs to me, they make very little use of. The fact I allude to, is, that the glands of Peyer are always involved in the disease; and I believe no fact in the entire range of pathology is better established than this, besides, it is equally a fact, that the liver is as infrequently found in an abnormal state in this disease as in any which produces death.

Writers, teachers, and practitioners appear to loose sight of this fact, and invariably recommend the use of mercury in the treatment of the disease—some in every stage, and some in particular stages. However much I may revere the name of John Estin Cook, and admire his industry and genius, I have very little respect for the man who can see nothing but disordered liver in every case of sickness he is called upon to treat; I, however, am proud to honor any man who gives mercury or any other remedy properly; but this idea of giving mercury for everything, is what has brought more reproach upon Western medical men than any other one thing, and I believe is the ground-work of the success of quacks and irregulars who now infest the West and the regular profession, hanging like gangrenous excrescences upon society.

Let us now look at this matter a moment. Physiologists tell us (and have told us from the days of Haller at least) that the bile is the natural purgative of the bowels, and that this effect is due more to its acrid, irritating qualities than to anything else. Diarrhœa is the thing to dread in the treatment of typhoid fever, because it is of the utmost importance to husband the strength of our patients by all possible means; and we need not say, the drainage produced by diarrhœa will soon exhaust the very element (vitality) which enters so largely into the curative process in this disease.

Is it too much to say, then, that the man who would give mercury with these facts before him, would be placing an obstacle in the way over which his patient will stumble into the grave? But I am met here by the stickler for the use of mercury, and told that we *must* unload the portal circle by increasing the action of the liver. *That* cannot be, because he has no evidence of the engorgement, nor that the liver has been remiss in any degree in the proper performance of its functions. I am perhaps met again by my pathological friend, who informs me that it is of no consequence if we do bring an irritating substance in contact with the mucous coat of the bowels, for the glands are entirely disconnected from the mucous lining, and, therefore, not subjected to any influence from irritation of this surface. I confess the anatomical fact, but beg to reply, that there will be a corresponding influence carried to the glands by irritation of the mucous surface in the same ratio that it is extended to the mucous or muscular coat by irritation of the glands, as is witnessed by the diarrhœa, which, as has been remarked, we have so much reason to dread: besides, it is only a few days in many instances before ulceration takes place, and then his bilious matter passing down would be quite a soothing and refreshing appliance. But I am now accosted by my friend, the Humeralist, and told that we must destroy the poison in the blood, and that in all fevers it is important to break up the plastic material in the blood. My cautious and considerate friend, I should reply, do you know that mercury will eliminate the poison, or by any process render it harmless? *I do not, neither do you.* Induction has not taught you it would, nor has observation. In regard to breaking up the plastic material of the blood, I could only wish in typhoid fever the blood contained more of it.

WM. M. CHAMBERS, M.D.

[N.B.—When the writer of the above asserts, that 'writers, teachers, and practitioners,' either lose sight of the diseased glands, or '*invariably* recommend the use of mercury in the treatment' of typhoid fever, we think him much mistaken—indeed, we know of no respectable teacher at the present day who does either.—*Editor of Joarnal.*]

